# The Relevance of Experimental Models in Assessing the Impact of Oxidative Stress on Intestinal Pathology

**DOI:** 10.3390/jcm14186569

**Published:** 2025-09-18

**Authors:** Cristian Dan Pavel, Cristina Gales, Irina Andreea Pavel, Carmen Lăcrămioara Zamfir

**Affiliations:** 1 Department of Morpho-Functional Sciences I, Faculty of Medicine, “Grigore T. Popa” University of Medicine and Pharmacy, University Street, No.16, 700115 Iasi, Romania; cristian-dan_g_pavel@d.umfiasi.ro (C.D.P.); cristina.gales@umfiasi.ro (C.G.); carmen.zamfir@umfiasi.ro (C.L.Z.); 2 Department of Surgery II, Faculty of Medicine, “Grigore T. Popa” University of Medicine and Pharmacy, University Street, No.16, 700115 Iasi, Romania

**Keywords:** experimental models, intestine, oxidative stress, glutathione

## Abstract

Oxidative stress is a state of imbalance between the process of producing and removing reactive oxygen species (ROS). With advancing age or in certain situations where oxidative stress cannot be combated, various pathologies such as inflammatory bowel diseases or neoplasia may occur. Over the past decade, a surge of intriguing discoveries has linked subtoxic levels of oxidative stress to key processes, including the maintenance of mucosal homeostasis, regulation of protective inflammation, and even the control of tissue wound healing. Given the complexity and limited understanding of oxidative mechanisms involved in human intestinal pathology, the relevance of experimental models becomes a critical consideration in efforts to elucidate these processes. Although diverse, none of these models fully replicate human digestive pathology; however, they remain valuable for developing new therapeutic strategies. This paper examines the main markers of oxidative stress and its mechanism and their impact on the intestinal tract, as well as the most widely used animal models that have contributed valuable insights into the pathogenesis of inflammatory bowel diseases (IBD).

## 1. Introduction

Oxidative stress occurs when antioxidant mechanisms cannot remove excess of reactive oxygen species (ROS) from the body and cause damage to proteins, lipids, and DNA. Over the past decade, a surge of intriguing discoveries has linked subtoxic levels of oxidative stress to key processes, including the maintenance of mucosal homeostasis, regulation of protective inflammation, and even the control of tissue wound healing. The production of ROS is an important factor in the pathogenesis of many inflammatory disorders, such as those in the gastrointestinal system, activating specific inflammatory signaling pathways and affecting immune reactivity. If the contribution of oxidative stress to the onset and progression of intestinal diseases is now broadly accepted, many of its underlying mechanisms and their relationships remain poorly understood [1,2].

Heightened oxidative stress directly affects intestinal morphology and permeability, together with gut microbiota, digestion, absorption, metabolic processes, and the immune response. In the gut, disruption of the mucosal barrier produces an activation of the innate immune system and an acute inflammatory reaction, initially located in lamina propria [1,2]. Elucidating the role of oxidative stress in digestive disorders not only facilitates the understanding of particular mechanisms involved but also is important in advancing new therapeutic strategies [3].

Given the current gaps in understanding these mechanisms, experimental models are essential tools for assessing oxidative stress-induced alterations in the intestine [3]. There are a lot of factors which should be considered when determining the relevance of experimental models. On this basis it can be concluded that no experimental model can fully replicate the diversity and complexity of the intestinal pathology, but they remain essential tools to investigate oxidative stress-induced alterations in intestinal structure and function [4].

Therefore, the aim of this narrative review is to synthesize the available evidence on experimental models of intestinal oxidative stress, highlighting their advantages, limitations, and the reasons why such models remain indispensable, despite the fact that no single experimental model can fully replicate the diversity and complexity of intestinal pathology.

## 2. Materials and Methods

The search was conducted in PubMed, Scopus, and ScienceDirect using the following keywords, experimental models, intestine, oxidative stress, glutathione, combined with Boolean operators (example: oxidative stress AND intestine AND experimental models). The last search was performed in March 2025.

**Figure 1:**
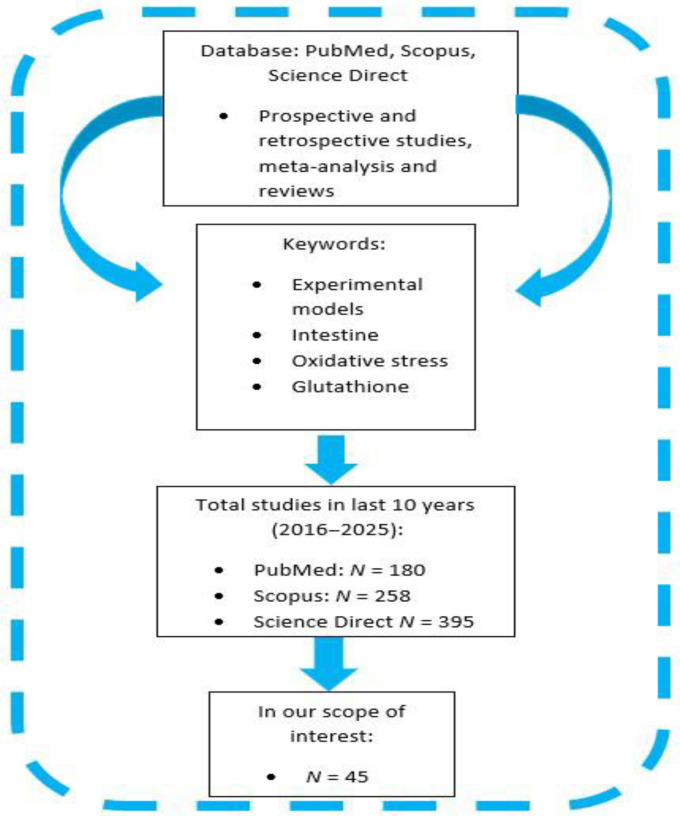
A schematic representation of the current study design.

We limited our search primarily to the last 10 years in order to capture the most recent developments in experimental models. However, we also included older, seminal studies that remain relevant for understanding fundamental mechanisms. Inclusion criteria included the following: prospective and retrospective studies, meta-analysis, and reviews. Exclusion criteria included the following: abstracts, conference proceedings, book chapters, editorials, and articles not addressing the scope of the review. Duplicates were removed manually. Our search resulted in 833 total references, and 45 papers were within our scope of interest. A schematic representation of the current study design is shown in Figure 1.

## 3. Intestinal Morphology and Redox Biology

As a prerequisite for understanding the role of experimental models, it is important to briefly review the main oxidative stress markers and antioxidant defense mechanisms.

### 3.1. Intestinal Morphology

The intestinal mucosa comprises a superficial layer of self-renewing epithelial cells and lamina propria with vascular, immune components. In the small intestine there are invaginations called Lieberkuhn’s crypts and protrusions into the lumen called differentiated cell villi. Crypts contain proliferative stem cells and Paneth cells responsible for innate immunity and antibacterial defense and for providing essential signals to intestinal stem cells [5].

The intestinal epithelium is made up of cells with an absorptive function (enterocytes), secretory cells (mucus-secreting goblet cells, Paneth cells-secreting antimicrobial peptides like lysozyme and α defensins, hormone-secreting enteroendocrine cells), and M-microfold cells that facilitate antigen presentation to lymphocytes from lamina propria. Enterocytes represent 80% of all cells, and enteroendocrine cells represent 1% of all epithelial cells. They originate in crypts, migrate to villi during differentiation, and then undergo spontaneous apoptosis and detach when they reach the tip of the villi after terminal differentiation [5].

Immune and inflammatory responses in the intestinal mucosa are marked by significant metabolic shifts in the tissue. These shifts involve high energy consumption and reduced oxygen availability (hypoxia) [6]. Such metabolic changes are driven by the recruitment of inflammatory cells, particularly neutrophils, other polymorphonuclear leukocytes (PMNs), and monocytes. A key feature of acute gut inflammation is the localized buildup of PMNs, known as crypt abscesses. Due to the substantial amounts of ROS generated by activated PMNs, crypt abscesses serve as critical hubs for reduction–oxidation (redox) signaling (a signaling response triggered by specific ROS) [7]. Resident immune cells in the gut, including intraepithelial lymphocytes and antigen-presenting cells (dendritic cells and macrophages), are primed to react to threats like bacterial and viral infections but also play a role in maintaining homeostasis through immune surveillance and promoting regulatory immune responses. Many of these cell types can either activate or bypass redox signaling, which has significant implications for mucosal homeostasis [8].

### 3.2. Intestinal Antioxidant Defense System

To combat oxidative stress, the body has an antioxidant defense system composed of endogenous enzymatic antioxidants and endogenous non-enzymatic antioxidants. Endogenous enzymatic antioxidants include superoxide dismutase (SOD), catalase (CAT), and glutathione peroxidase (GSH-Px). Endogenous non-enzymatic antioxidants include glutathione, thioredoxin (Trx), and irisin. Exogenous antioxidants are represented by essential nutrients and nutritional supplements [9]. Table 1 summarizes the mechanisms of action of endo- and exogenous antioxidants.

### 3.3. Glutathione Synthesis and Its Particular Role in the Gut

The intestinal epithelium, a component of mucosa, has a particular luminal distribution, which makes it a significant protective structure against extraluminal diverse factors. Glutathione, a potent antioxidant abundantly present in intestinal epithelial cells, plays a central role in maintaining mucosal integrity. Glutathione is a tripeptide (g-Glu-Cys-Gly) present in high concentrations in tissues, essential for the proper functioning and maintenance of the structural integrity of the gastrointestinal tract. Epithelial cells in the jejunum and colon are dependent on the presence of GSH, and a deficiency could affect the gastrointestinal tract at this level [3]. The GSH/GSSG (glutathione disulfide) redox couple provides a redox environment that allows maintenance of gut microbiota, adequate nutrient absorption, reversal of oxidant-induced epithelial damage, modulation of intestinal cell transformation, and apoptosis [8].

GSH is essential for the proper functioning and maintenance of the structural integrity of the intestinal tract. Epithelial cells in the jejunum and colon are dependent on the presence of GSH, and a deficiency of GSH could affect the intestinal tract at this level [3].

GSH concentrations are much higher in the gastric glands, conferring protection against the effects of stomach acid. Inflammation induced by *Helicobacter pylori* infection results in damage due to the production of ROS, exceeding the protective capacity of gastric mucosal cells and glutathione. Regulation of glutathione concentration prevents injury caused by *Helicobacter pylori* infection, showing the importance of maintaining the relative balance between pro- and antioxidants [25]. Intestinal GSH concentrations are in the millimolar range (2–10 mmol/L). At the intracellular level it is found in the reduced thiol form, and the oxidized form is glutathione disulfide (GSSG) and is associated with oxidative stress [26].

GSH in the intestinal epithelium is synthesized in the cytosol via two ATP-dependent steps: glutamate-cysteine ligase forms γ-glutamyl-cysteine, and glutathione synthetase adds glycine to complete the tripeptide, while glutathione reductase reduces oxidized glutathione back to GSH using NADPH. At the brush border, γ-glutamyl transpeptidase and a dipeptidase degrade luminal GSH into amino acids that enterocytes re-use for GSH synthesis; together, these processes constitute the γ-glutamyl cycle [4].

Diet and bile provide luminal GSH; bile secretion into the duodenum supplies roughly half of hepatic GSH output. Enterocytes absorb intact GSH across the apical membrane through transporters that operate independently of GSH synthesis and are stimulated by monovalent cations. Any luminal GSH not taken up is hydrolyzed by apical γ-glutamyl transpeptidase, even at low concentrations [26].

Within the epithelium, the GSH/GSSG redox couple sustains a reducing environment that supports nutrient absorption, preserves the mucus layer, and modulates proliferation, differentiation, and apoptosis of enterocytes [4]. Luminal GSH reduces dietary disulfides, conjugates xenobiotics, chelates metals, and contributes to mucus fluidity, thereby protecting the mucosal surface [26]. Adequate GSH is particularly important for active calcium transport: depletion of mucosal GSH leads to epithelial degeneration and impaired Ca^2+^ absorption, whereas oral GSH or its monoester restores both epithelial integrity and transport capacity. GSH depletion or oxidant-rich diets trigger oxidative and nitrosative stress and apoptosis in enterocytes, diminishing nutrient absorption, while antioxidants such as quercetin, naringin, ursodeoxycholic acid, melatonin, and glutamine replenish GSH and normalize these functions [4].

At the brush border of the intestinal epithelium, a cysteine/cystine shuttle works alongside the γ-glutamyl cycle to control luminal thiol/disulfide balance, and within enterocytes, reduction in cystine and continuous GSH synthesis keep both the cysteine/cystine and GSH/GSSG redox couples in the proper range. This finely tuned system enables intestinal epithelial cells to cope with constant exposure to luminal oxidants, maintain barrier integrity, and support efficient nutrient uptake and detoxification [26].

### 3.4. Redox Signaling in the Gut

The redox state of the intestinal tract is determined by the balance between antioxidants and oxidants. When this balance is disrupted—either by an excess of oxidants or a deficiency of neutralizing antioxidants—the tissue experiences oxidative or nitrosative stress. In the intestinal tract, various ROS, such as superoxide (O_2_•^−^) and hydroxyl radicals (•OH), along with non-radicals like hypochlorous acid (HOCl) and hydrogen peroxide (H_2_O_2_), are produced by epithelial cells, endothelial cells, and immune cells to support mucosal defense [27].

The enzymatic use of molecular oxygen (O_2)_ within the intestinal mucosa facilitates redox signaling, creating spatial and dynamic patterns of oxygen availability. In healthy intestinal mucosa, there is a steep oxygen gradient between the well-vascularized mucosa and the anoxic lumen. As a result, cells near crypt stem cells typically experience higher partial pressures of oxygen (pO_2_; ~100 mmHg) compared to lumen-facing epithelial cells (<10 mmHg) [28].

## 4. Oxidative Stress Involved in Inflammation-Based Intestinal Diseases

### 4.1. Collateral Damage to Digestive Tissues Induced by Reactive Oxygen Species

When antioxidant defenses fail, excess ROS damage proteins, lipids, and DNA, impairing intestinal morphology, barrier integrity, metabolism, and immune regulation [2]. Major ROS sources in the intestinal tract include xanthine oxidase, diamine oxidase, aldehyde oxidase, and NADPH oxidase [29]. Excessive ROS from pro-inflammatory cells drive chronic intestinal inflammation, linked to reduced GSH and increased GSSG. Patients with inflammatory bowel disease have lower mucosal GSH and decreased T lymphocyte proliferation in the lamina propria. Bacterial ROS can alter Trx status and inhibit NF-κB activity, while microbiota composition affects pathogen resistance. Chronic oxidative stress and inflammation raise cancer risk, with elevated Trx1 and Grx3 promoting colorectal cancer growth, whereas reduced Grx3 inhibits tumor progression [26].

Tight junction proteins are crucial for maintaining the intestinal barrier and regulating permeability; their disruption leads to gut barrier damage and increased permeability, contributing to diseases like inflammatory bowel disease [30]. Redox balance is vital for intestinal flora, with GSH in the lumen supporting mucus fluidity, detoxifying xenobiotics, and metabolizing peroxidized lipids [31]. Impaired redox balance weakens antimicrobial defenses, prolongs immune activation, and alters immune responses, leading to incomplete pathogen removal and chronic inflammation that can cause complications like fibrosis and neoplasia [1]. Both Crohn’s disease and ulcerative hemorrhagic colitis involve chronic inflammation due to inadequate inflammatory responses in genetically predisposed individuals, marked by elevated ROS and reduced antioxidants. ROS further enhances pro-inflammatory factors, worsening tissue damage and disease progression [25].

Both excess and deficiency of ROS contribute to inflammatory bowel disease (IBD). Genome-wide association studies reveal that risk factors vary among individuals, not solely linked to ulcerative colitis or Crohn’s disease. For instance, altered oxygen levels in chronic granulomatous disease significantly increase IBD risk [32]. Elevated oxidative stress from intestinal inflammation leads to tissue damage, impaired absorption, gut barrier dysfunction, and altered motility [28]. Nutrient malabsorption occurs after ischemia–reperfusion injury and in IBD [33]. Impaired absorption in the colon’s epithelial tissue results in diarrhea [34]. Extensive ROS-induced damage and increased immune mediators like TNF-α and IFN-γ enhance mucosal permeability by affecting tight junctions [35].

Increases in vascular permeability can precede epithelial permeability during active mucosal inflammation. Tolstanova et al. demonstrated that early endothelial injury causes perivascular edema and epithelial hypoxia, stabilizing hypoxia-inducible factors in the mucosa [36]. Additionally, intestinal motility is influenced by redox-sensitive mechanisms; Brown et al. found that glial cell-derived nitric oxide (NO) mediates enteric neuron death during active colitis, impacting connexin activity. Furthermore, exposure to microbial lipopolysaccharides (LPS) impairs motility by generating excess ROS and reactive nitrogen species (RNS) in submucosal smooth muscle cells [37]. These findings highlight the tissue damage linked to oxidative stress during gut inflammation.

### 4.2. Toxin/Drug Exposure

Oxidative stress plays a role in inflammation-driven intestinal diseases. The involvement of ROS in various GI dysfunctions, as well as their dual roles in cancer promotion and suppression, will be explored to deepen our understanding of inflammation-based GI disorders [38].

Chemotherapeutic agents produce high levels of ROS, which play a significant role in the pathology of drugs like anthracyclines and platinum-based compounds. While these agents use ROS to kill cancer cells, they also cause common side effects, including intestinal toxicity and mutagenesis. Excess ROS can lead to severe issues such as mucosal damage, loss of epithelial cells and tight junction proteins, microbiota imbalance, and enteric neuropathy. A major side effect of chemotherapy is mucositis, which results in significant gastrointestinal inflammation, nausea, diarrhea, bleeding, and abdominal pain [39].

Radiotherapy for abdominal and pelvic cancers often leads to gastrointestinal complications, with oxidative stress being a key mechanism for cancer cell death [40]. Prolonged NSAID use carries a high risk of gastrointestinal issues, including peptic ulcers, as NSAID metabolites may induce ROS generation [41]. Excessive alcohol consumption also contributes to oxidative stress through byproducts of ethanol metabolism and NAD depletion [39].

### 4.3. Ischemia–Reperfusion and Postoperative Injury

Ischemia–reperfusion injury is a major issue in ischemic syndromes and solid organ transplantation, occurring in various tissues upon reoxygenation. This injury is primarily driven by oxidative stress, which activates an immune response in the affected tissue [42]. The gut is particularly susceptible due to its ability to generate numerous free radicals [39].

Postoperative ileus, or paralytic ileus, is characterized by reduced intestinal activity following bowel manipulation during surgery. Research indicates that it is a complex condition involving initial activation of cells in the muscularis propria [43].

### 4.4. Congenital Pathologies

Genetic intestinal disorders may be linked to oxidative stress, though the complexity of mutations requires further investigation. For instance, Triple-A syndrome has been noted, and recent reports suggest oxidative stress may play a role in Hirschsprung’s disease [44,45].

### 4.5. Inflammation and Infection

Oxidative stress is crucial for normal immune functions, as free radicals help eliminate bacteria and diseased cells and activate inflammatory pathways. However, non-specific free radical signaling can cause tissue damage and lead to inflammation, a phenomenon well-studied in IBD [46,47].

Necrotizing enterocolitis (NEC) is a life-threatening intestinal condition that primarily affects premature, low-birth-weight infants. The pathogenesis of NEC is closely linked to tissue oxygenation levels. This multifactorial disorder involves immune stimulation in the preterm gut during colonization by intestinal flora [48].

Oxidative stress also seems to be involved in parasitic infections of the gut, such as Chagas disease which is caused by infection with the parasite *Trypanosoma cruzi.* In this case, megacolon or megaesophagus only occurs in a limited number of patients [49].

### 4.6. Cancer

Intestinal inflammation promotes oxidative stress and free radical accumulation, which accelerates carcinogenesis. Consequently, patients with IBD have a markedly increased risk of colorectal cancer [50,51,52]. Similarly, Barrett’s esophagus is linked to oxidative stress, which can lead to esophageal adenocarcinoma [53].

Table 2 summarizes information on oxidative stress in intestinal pathology.

## 5. Experimental Models of Inflammatory Bowel Diseases

Because oxidative mechanisms in human intestinal pathology remain insufficiently understood, experimental models are essential for clarifying their role.

A key feature is the remarkable diversity of experimental models and species used, reflecting the ongoing search for reliable systems to assess the impact of oxidative stress in the digestive tract.

### 5.1. Mice

The most used species in experimental models for studying oxidative stress in intestinal system is the mouse.

#### 5.1.1. DSS- and TNBS-Induced Colitis Models

Most patients with chronic granulomatous disease exhibit symptoms characteristic of inflammatory bowel disease. A complicating factor in research in this area is the use of mouse models to differentiate the roles of phagocyte-derived ROS versus those originating from mucosal sources. In studies using 2,4,6-trinitrobenzenesulfonic acid (TNBS) to model colitis, researchers found that Nox2^−/−^ mice developed significantly more severe colitis, as evidenced by weight loss, increased intestinal permeability, and an inability to resolve inflammation, compared to wild-type controls [6]. Conversely, Bao et al. used the same Nox2^−/−^ mice to study dextran sulfate sodium (DSS)-induced colitis and found no difference in weight loss or disease severity compared to wild-type controls, concluding that reduced tissue damage was linked to a diminished oxidative response [65].

One potential explanation for the discrepancies between these studies is the nature of the models used to assess the relative importance of NOX phagocytosis. DSS-induced colitis models involve the denudation of epithelial cells, beginning with apical mucus erosion and epithelial apoptosis, leading to an immune infiltrate. In this context, it could be argued that phagocyte-derived ROS may not significantly contribute to the denudation of colonic epithelia; therefore, only interventions affecting epithelial viability or turnover would have a notable impact. In contrast, TNBS-induced colitis models involve pre-sensitization of the host immune system. DSS results in progressive tissue damage that extends from the rectum and is associated with gradual weight loss. By contrast, TNBS-treated animals exhibit rapid weight fluctuations and discontinuous lesions, often with relatively preserved epithelia. Furthermore, the immune infiltrates and inflammatory mediators differ significantly between the models. Thus, DSS serves as a model of tissue deterioration, whereas TNBS represents a model of acute to chronic inflammation and resolution. Consequently, it is plausible that the Nox2^−/−^ mutation does not lead to increased mucosal damage [66].

#### 5.1.2. Oxazolone-Induced Colitis Model

Oxazolone is a haptenizing agent commonly used to induce colitis in mice for studying the pathological processes involved in ulcerative colitis. This colitis model triggers a Th2 cell-mediated immune response and has been shown to closely resemble human ulcerative colitis, exhibiting mucosal membrane inflammation, epithelial microulcerations, and histopathological changes in the distal colon. The cellular and immune responses, as well as the cytokine secretion profiles in oxazolone-induced colitis, differ from those observed in TNBS colitis. Specifically, oxazolone-induced colitis is marked by significant IL-13 production from natural killer T (NKT) cells in the lamina propria CD4, rather than the typical IFN-γ production from conventional CD4+ T cells [67].

#### 5.1.3. Adoptive T-Cell Transfer Experimental Model

In 1990, an adoptive T-cell transfer system was established to induce colitis in immunodeficient mice, significantly advancing the concept of “regulatory T cells” [68]. A major breakthrough in IBD research occurred in 1993 with the discovery of spontaneous colitis in three types of knockout (KO) mice: interleukin (IL)-2 KO, IL-10 KO, and T-cell receptor (TCR) KO [69]. Since then, over 40 different genetically engineered KO mouse strains and strains with congenital mutations that spontaneously develop colitis and/or ileitis have been identified [67].

#### 5.1.4. IL-10 Knockout Model

The development of murine models to study the pathophysiology of IBD continued with the creation of a genetically engineered model using IL-10-deficient mice [70]. This model underscored the significant anti-inflammatory role of IL-10. Additionally, genetic polymorphisms at the IL-10 locus have been linked to the development of Crohn’s disease (CD) and ulcerative colitis (UC). IL-10 is a well-known regulatory cytokine and a key susceptibility gene for IBD (both UC and CD). IL-10 KO mice, which lack the IL-10 gene, spontaneously develop colitis after three months of age [71].

#### 5.1.5. SAMP1/YitFc Colitis

The SAMP1/YitFc mouse strain serves as a model for CD-like ileitis, making it ideal for investigating the pathogenesis of chronic intestinal inflammation. Unlike most animal models of colitis, the ileal phenotype specific to SAMP1/YitFc mice occurs spontaneously, without any genetic, chemical, or immunological manipulation. Furthermore, SAMP1/YitFc mice exhibit remarkable similarities to human disease in terms of disease localization, histological features, incidence of extraintestinal manifestations, and response to conventional therapies [72].

#### 5.1.6. Microbiome-Induced Mouse Models

Using culture-independent molecular methods, researchers have identified over 1000 species of bacteria residing in the gastrointestinal tract, with the collective genome of gut microbes containing approximately 100 times more genes than the human genome. In patients with IBD, there is a notable anti-inflammatory effect associated with decreased bacterial diversity and an increase in pro-inflammatory bacteria compared to healthy individuals. The most consistent changes include reduced gut microbiota diversity and lower levels of Firmicutes [67]. Table 3 summarizes various experimental models of inflammatory bowel disease in mice.

### 5.2. Guinea Pigs

Guinea pigs are commonly used to study the enteric nervous system due to their neurochemical similarities to humans with IBD. Their sensitivity to sensitizing substances makes them ideal for immunological research, particularly in DSS-induced colitis. However, their high care costs limit their utility [93].

### 5.3. The New Zealand White Rabbit

New Zealand white rabbits facilitate endoscopic biopsies due to their larger size. Inducing colitis with DSS allows observation of disease progression from acute to chronic stages. They are recommended for studies requiring endoscopic procedures [93].

### 5.4. Dogs

Dogs are frequently used in drug discovery and safety evaluation. Canine idiopathic IBD shares similarities with human IBD, involving immune, genetic, and environmental factors. Despite their usefulness as models, the high costs associated with using dogs can be a drawback [94].

### 5.5. Pigs

Pigs share comparable nutritional and immunological features with humans, making them a promising model for IBD. DSS-induced colitis has been successfully reproduced in young piglets, aiding research on mucosal immunity and dietary effects. Pigs also exhibit chronic inflammation patterns similar to human UC, despite the high costs involved [95,96,97].

### 5.6. Monkeys

Primates, such as rhesus macaques and cotton-top tamarins, are crucial for biomedical research due to their biological similarity to humans. They often show spontaneous enteritis, resembling human IBD. Studies reveal that many macaques share differentially expressed genes with humans and mice, making them valuable for IBD research, although their limited availability poses challenges [98].

### 5.7. Zebrafish

Zebrafish, with 87% genetic similarity to humans, are low-cost models for studying IBD. Their transparency allows dynamic observation of disease development. TNBS- and DSS-induced intestinal inflammation in zebrafish reflects changes in gut microbiota and immune responses similar to mammalian IBD. While their differences from humans limit direct applicability, they provide valuable insights into pathogenesis and drug testing [99,100,101,102].

A study conducted by Rastogi et al., showed that the ability of the GSH system to respond and regenerate after oxidation changes with developmental stage. Zebrafish embryos are resistant to oxidative stress 18 h after fertilization. After hatching, embryos become more sensitive to the action of pro-oxidizing factors. This is due to changes in GSH concentration during embryo development [103].

Glutathione is the most abundant and most commonly studied antioxidant in zebrafish when assessing oxidative stress. Oxidative stress results in decreased glutathione concentration. Zebrafish glutathione concentration decreases in response to multiple substances such as arsenic, atrazine, cadmium, chromium, cadmium sulfate, silver nanoparticles, titanium dioxide nanoparticles, bisphenol A, nonylphenol, and ionizing radiation. Glutathione is used in order to neutralize ROS causing an increase in GSSG concentration. Assessment of both GSH and GSSG allows calculation of redox status (ratio of GSSG to GSH) being a biomarker of oxidative stress. There are different concentrations of GSH and GSSG depending on the stage of embryonic development. GSH dynamics are important for maintaining redox homeostasis and suggest that there are stages at which embryos are susceptible to oxidative stress [104].

Many xenobiotic agents can induce oxidative stress, with the risk of overwhelming the body’s antioxidant defense system and damaging proteins, lipids, and genetic material. Zebrafish is a useful experimental model to study the toxicity of different xenobiotic agents and oxidative stress by having an extensive antioxidant system [104].

Zebrafish are extensively utilized as an animal model to investigate human development, genetics, and disease. Recently, they have gained prominence as a model for exploring the regulatory mechanisms of ion homeostasis in body fluids. Hormonal control plays a crucial role in regulating ions within these fluids. Several hormones, such as parathyroid hormone (PTH) and vitamin D, are well known for maintaining Ca^2+^ homeostasis in mammals. Recent studies have examined the roles of these calciotropic hormones in zebrafish. The expression and secretion of calciotropic hormones in zebrafish are influenced by extracellular Ca^2+^ levels. The calcium-sensing receptor (CaSR) located in specific endocrine organs detects these extracellular Ca^2+^ levels and modulates the expression and secretion of mammalian calciotropic hormones. Recent research has also defined the effects of CaSR on hormone regulation in zebrafish [105].

### 5.8. Drosophila melanogaster

*Drosophila melanogaster* serves as a valuable model for studying intestinal diseases due to its human-like anatomical features. It helps investigate the regulatory pathways of innate immunity related to IBD, such as the balance between microbe-induced epithelial cell damage and stem cell repair via Toll signaling. This model has also been used to explore the effects of Epstein–Barr virus DNA on IBD. Future research could leverage *Drosophila* for large-scale genetic screening to uncover processes related to intestinal cell proliferation, differentiation, and function, potentially offering new insights into the mechanisms of intestinal diseases [106,107,108].

### 5.9. Worms

*Caenorhabditis elegans* shares significant genomic homology with mammals and is transparent during growth, allowing for easy cell visualization. While it enables the study of bacterial interactions with intestinal cells, its applicability to IBD research is limited due to the absence of immune and circulatory systems. Nonetheless, worms are valuable for understanding inflammation mechanisms in the human gut and screening new drugs for IBD treatment [109,110,111]. Table 4 summarizes different experimental models of inflammatory bowel disease.

## 6. Conclusions

Oxidative stress is central to the onset and progression of intestinal disease. Experimental models remain indispensable for identifying biomarkers, elucidating mechanisms, and testing potential therapies. Across models, oxidative stress is commonly assessed by the GSH/GSSG redox couple and antioxidant enzymes (SOD, CAT), together with global ROS-related readouts where applicable. Although no single model fully replicates human intestinal diseases, chemically induced colitis models are useful for acute inflammation, genetically modified animals provide insights into chronic mechanisms, and cell culture systems allow mechanistic exploration but lack systemic relevance. The choice of model should depend on the research question. Future studies should standardize redox panels and reporting, validate findings across models/species, and expand human-relevant platforms that better reproduce the complexity of human intestinal pathology.

## Figures and Tables

**Table 1:** Published studies that analyze the mechanism of actions of endogenous and exogenous antioxidants.

Substance	Mechanism of Action	Author and Year
Superoxide dismutase	Superoxide dismutase converts superoxide radicals to hydrogen peroxide and molecular oxygen, reducing oxidative stress.	Rampon et al., 2018 [10]
Superoxide dismutase	Superoxide dismutase inhibits the production of pro-inflammatory cytokines, reducing colitis symptoms.	Liang et al., 2022 [11]
Glutathione (GSH)	Glutathione protects against reactive oxygen and nitrogen species, playing a crucial role in maintaining redox homeostasis and regulating immune responses.	Vašková et al., 2023 [12]
Glutathione peroxidase (GPx)	Glutathione peroxidase regulates Th17 lymphocyte differentiation, promoting immune regulation and antioxidant defenses.	Morris et al., 2022 [13]
Catalase	Low catalase levels reduce autophagy-dependent cell death, which may exacerbate conditions like Crohn’s disease.	Iborra et al., 2022 [14]
Vitamin C	Vitamin C improves neutrophil chemokinesis and chemotaxis, enhancing immune response.	Elste et al., 2017 [15]
Vitamin E	Vitamin E inhibits reactive oxygen species production during fat oxidation and free radical reactions.	Rizvi et al., 2014 [16]
Zinc	Zinc deficiency increases pro-inflammatory cytokines like TNF-α and IL-6, impacting immune function.	Gammoh et al., 2017 [17]
Selenium	Selenium affects immune cells by influencing antioxidant functions and signaling pathways in cells like NK cells and T lymphocytes.	Avery et al., 2018 [18]
Betacarotene	Betacarotene scavenges superoxide radicals and quenches singlet oxygen, reducing oxidative stress.	Miazek et al., 2022 [19]
Betacarotene	Betacarotene reduces inflammation by downregulating the TLR4 signaling pathway.	Cheng et al., 2021 [20]
Flavonoids	Flavonoids inhibit the NF-κB cascade, which is involved in inflammatory responses.	Li et al., 2020 [21]
Flavonoids	Flavonoids chelate metal ions, uptake reactive oxygen species, and stimulate detoxification enzymes.	Slika et al., 2022 [22]
Short-chain fatty acids (e.g., butyrate) produced by gut bacteria	Certain gut bacteria ferment dietary fiber to produce short-chain fatty acids; butyrate stimulates mitochondrial biogenesis, improves respiratory capacity, and activates antioxidant enzyme activity, enhancing antioxidant defenses and modulating redox signaling.	Zhao et al., 2023 [23]
Metal-binding proteins (ceruloplasmin, ferritin)	Ceruloplasmin oxidizes ferrous iron to ferric iron and binds it to transferrin for safe transport, while ferritin stores iron within enterocytes. By sequestering free iron, these proteins limit iron-mediated production of reactive oxygen species.	Loveikyte et al., 2023 [24]

**Table 2:** Summarized table of information on oxidative stress in intestinal pathology.

Pathology	Mechanism of Action	Author and Year
Drug/Toxin Exposure—Chemotherapy	Chemotherapy causes excessive production of ROS, leading to intestinal side effects such as mucositis. ROS and inflammatory mediators contribute to tissue damage in the gut.	McQuade et al., 2016 [54] Al-Asmari et al., 2016 [55]
Drug/Toxin Exposure—Radiotherapy	Radiation reduces the body’s enzymatic antioxidant defenses, impairing the ability to neutralize oxidative stress, which may cause long-term damage to the gut.	Musa et al., 2019 [56]
Drug/Toxin Exposure—NSAIDs	NSAIDs like indomethacin increase mitochondrial superoxide and xanthine oxidase levels in colonic epithelial cells, leading to higher superoxide production and oxidative stress.	Nagano et al., 2012 [57]
Drug/Toxin Exposure—Alcohol	Alcohol metabolites like ethanol and acetaldehyde cause oxidative stress in epithelial cells, disrupting tight junction integrity and raising superoxide levels, leading to increased gut permeability.	Samak et al., 2016 [58]
Ischemia–Reperfusion	During intestinal ischemia–reperfusion, xanthine dehydrogenase converts to xanthine oxidase, which produces free radicals upon reoxygenation, leading to oxidative stress and tissue injury.	Sasaki et al., 2007 [59]
Postoperative Injury	Oxidative stress can occur independently of inflammation after tissue injury, possibly promoting further inflammation in postoperative injury scenarios.	Matsumoto et al., 2018 [60]
Congenital Pathologies—Triple-A syndrome	In Triple-A syndrome, mutations in the AAAS gene disrupt the redox balance, increasing susceptibility to oxidative stress-induced cell death, particularly in tumors.	Prasad et al., 2014 [44]
Congenital Pathologies—Hirschsprung’s disease	Oxidative stress during development impairs the formation of the enteric nervous system, contributing to congenital pathologies like Hirschsprung’s disease.	Zhou et al., 2022 [45]
Inflammation—Inflammatory bowel disease	Oxidative stress plays a significant role in IBD, leading to chronic inflammation and tissue damage in colitis models.	Sahakian et al., 2021 [61]
Inflammation—Necrotizing enterocolitis	Elevated oxidant levels are observed in patients with necrotizing enterocolitis, contributing to oxidative damage and inflammation in the gut.	Aydemir et al., 2011 [62]
Infection	The MRPS18B P260A variant in Chagas megaesophagus patients affects mitochondrial function and induces nitro-oxidative stress, exacerbating the disease.	Silva et al., 2022 [49]
Cancer	In colorectal cancer, free radicals from various sources induce oxidative stress, leading to genomic instability and transforming normal colon cells into dysplastic and neoplastic cells.	Carini et al., 2017 [63]
Cancer	In Barrett’s esophagus, oxidative stress is indicated by high levels of peroxynitrite, superoxide, and glutathione, which contribute to tissue damage and may lead to esophageal cancer.	Jiménez et al., 2005 [64]

**Table 3:** Summarized table of evolution and diversity of experimental murine models of inflammatory bowel disease.

Species/Strain	Induction/Intervention Method	Oxidative Stress Markers Assessed	Main Outcomes	Limitations	Author and Year
*Mouse* (various; commonly C57BL/6/BALB/c)	DSS-induced colitis	Not reported	Epithelial denudation, barrier disruption, acute inflammation with increased ROS and lipid peroxidation	Models acute injury; limited chronicity; dosing/time dependence	Chassaing et al., 2014 [73]
*Rat/Mouse*	TNBS-induced colitis	MPO (Myeloperoxidase)	Th1-mediated colitis; oxidative injury with elevated ROS/MPO and mucosal damage	Sensitization-dependent; variability in severity; solvent effects	da Silva et al., 2010 [74]
*Mouse (BALB/c)*	Oxazolone-induced colitis	Not reported	Th2/NKT-driven colitis mimicking UC features; mucosal inflammation, microulcerations	Primarily distal colon; model reproducibility varies	Kojima et al., 2004 [75]
*Mouse (Rag2^−/−^ recipients)*	Adoptive CD4+ T-cell transfer	Not reported	Chronic colitis via Th1 effector responses; sustained inflammation	Requires immunodeficient hosts; labor-intensive	Corazza et al., 1999 [76]
*Mouse (C57BL/6J*; 6 to 8 weeks; female)	Heavy-ion radiation-induced intestinal oxidative stress in mice	Intracellular ROS, mitochondrial superoxide, NADPH oxidase activity, mitochondrial membrane potential, DNA oxidative damage, mitotic activity	^56Fe radiation caused persistent increases in mitochondrial ROS, enhanced NADPH oxidase activity, mitochondrial dysfunction, and oxidative DNA damage in intestinal epithelial cells compared with γ-irradiation	Only one post-exposure time point (1 year); no assessment of early dynamic changes; applicability limited to radiation-induced oxidative stress	Datta et al., 2012 [77]
*Mouse (SAMP1/YitFc)*	Spontaneous mutation model	Not reported	Spontaneous CD-like ileitis; chronic inflammation; extraintestinal features	Strain-specific; limited availability	Kosiewicz et al., 2001 [78]
*Mice (TNBS model)*	Colitis induced with TNBS; treatment with Protocatechuic Acid (PCA)	GSH, GSSG/GSH ratio, SOD, CAT, Nrf2 expression	TNBS colitis decreased antioxidant defenses (↓ GSH, ↓ SOD, ↓ CAT, ↓ Nrf2). PCA treatment restored antioxidant enzyme activity, improved GSH balance, and reduced oxidative stress and inflammation	Only one antioxidant compound tested; short-term study, no long-term outcomes assessed	Crespo et al., 2017 [79]
*Mouse (IL-7 transgenic)*	Genetic—IL-7 overexpression	Not reported	Persistence of colitogenic CD4+ memory T cells; chronic colitis	Immune-niche specific; not a pure OS model	Nemoto et al., 2007 [80] Nemoto et al., 2009 [81] Nemoto et al., 2011 [82] Nemoto et al., 2013 [83]
*Mouse (IEC STAT3/IL-22 axis)*	Genetic—IL-22 deficiency/impaired STAT3	Not reported	Defective mucosal healing; worsened colitis	Focused on repair pathways; OS not primary endpoint, but secondary effect	Pickert et al., 2009 [84]
*Mouse (XBP1 in IECs)*	Genetic—XBP1 disruption (ER stress)	Not reported	Paneth/goblet cell defects; inflammation driven by ER stress	Emphasizes ER stress; OS not primary endpoint, but secondary effect	Kaser et al., 2008 [85] Adolph et al., 2013 [86]
*Mouse (T-bet^−/−^Rag2^−/−^; TRUC)*	Genetic—TRUC model	Not reported	Communicable UC-like colitis; microbiota-immune interactions	Complex immune/microbiota interplay; OS not primary endpoint, but secondary effect	Garrett et al., 2007 [87]
*Mouse (Mdr1a^−/−^)*	Genetic—Mdr1a^−/−^ colitis	Not reported	Barrier dysfunction; bacterial translocation; chronic colitis	Transporter-specific; strain housing effects	Panwala et al., 1998 [88] Resta-Lenert et al., 2005 [89] Collett et al., 2005 [90]
*Mouse (Muc2^−/−^)*	Genetic—Muc2^−/−^ deficiency	Not reported	Mucus barrier loss; spontaneous colitis; tumor risk	Strong barrier phenotype; OS readouts often indirect	Velcich et al., 2002 [91] Van der Sluis et al., 2006 [92]

Arrows (↓) indicate direction of change: ↓ = decrease.

**Table 4:** Summarized table of experimental models of inflammatory bowel disease.

Species/Strain	Induction/Intervention Method	Oxidative Stress Markers Assessed	Main Outcomes	Limitations	Author and Year
*Guinea pig*	DSS colitis + curcumin C-SLNs	LPO, protein carbonyl, MPO	↓ leucocyte infiltration, ↓ OS, ↓ TNF-α; preserved colonic structure	Species cost; limited resources	Sharma et al., 2019 [112]
*Guinea pig*	DSS colitis + *B. vulgatus* 7K1	Not reported	Microbiota modulation; ↓ colitis severity	Probiotic strain specificity; OS not a primary endpoint (secondary/indirect effect)	Li et al., 2021 [113]
*New Zealand White rabbit*	DSS colitis; endoscopic monitoring	Not reported	Enables longitudinal endoscopic/bioptic assessment	OS markers not detailed	Lei et al., 2019 [114]
*Dog* (spontaneous IBD)	Clinical evaluation	Not OS; fecal canine calprotectin (cCP)	cCP as non-invasive inflammation biomarker	OS markers not measured	Otoni et al., 2018 [115]
*Pig / Piglet*	DSS colitis; diet interventions	Mostly inflammatory gene expression; OS not reported	UC-like features; dietary effects on mucosal immunity	High cost; OS readouts variable	Nielsen et al., 2020 [95]
*Rhesus macaque* (spontaneous enteritis)	Natural disease	Not reported	Immune expression profiles overlap with human IBD	Limited availability; ethics; OS not a primary endpoint (secondary/indirect effect)	Wang et al., 2021 [98]
*Zebrafish* (larvae/juvenile)	DSS/TNBS; toxicants; extracts	Not reported	Mirrors microbiota/immune shifts; neuronal changes in colitis	Developmental stage-dependent	Uyttebroek et al., 2020 [100]
*Zebrafish*	Chemical; viral components	Not reported	Innate immunity and epithelial repair pathways in GI inflammation	Invertebrate; translational limits	Hanyang et al., 2017 [102]
*Zebrafish*	Bacteria–epithelial interactions	SOD	Conserved TFs/pathways; drug screening utility	No adaptive immunity/circulation	Li et al., 2022 [116]
*Drosophila melanogaster*	Chemical; viral components	Not reported	Innate immunity and epithelial repair pathways in GI inflammation	Invertebrate; translational limits	Madi et al., 2021 [108]
*Caenorhabditis elegans*	Bacteria–epithelial interactions	Not reported	Conserved TFs/pathways; drug screening utility	No adaptive immunity/circulation	Haerty et al., 2008 [109]

Arrows (↓) indicate direction of change: ↓ = decrease.

## Data Availability

Data sharing is not applicable to this article.
